# Ansa Cervicalis to Marginal Mandibular Nerve Transfer, a Feasible Option to Improve Lower Lip Function in Facial Nerve Paralysis

**DOI:** 10.1177/22925503251386749

**Published:** 2025-10-30

**Authors:** Marieta Van der Vyver, Eva Lindell-Jonsson, Kristin N. L. Bell, Ashvir Singh, Shamir P. Chandarana, Alan Robertson Harrop, Robert D. Hart, Wayne T. Matthews, Ashley Hinther, Charles David McKenzie, Jennifer K. L. Matthews, Christiaan Schrag

**Affiliations:** 1 Section of Plastic Surgery, 2129 University of Calgary, Calgary, Canada; 2 Department of Physiotherapy, 3146 Alberta Health Services, Calgary, Canada; 3 Section of Otolaryngology, 2129 University of Calgary, Calgary, Canada

**Keywords:** ansa cervicalis, marginal mandibular, nerve transfer, facial nerve paralysis, anse cervicale, nerf mandibulaire marginal, transfert de nerf, paralysie du nerf facial

## Abstract

Facial nerve palsy affecting the lower face may compromise essential functions such as speech and eating. The associated facial asymmetry can contribute to social isolation. Facial nerve reconstruction using a combination of interposition nerve grafts and/or nerve transfers offers significant improvement. However, these options are limited by insufficient donor nerves and unwanted synkinesis if a single donor nerve is used for the entire hemi-face. To ameliorate both limitations, we perform an ansa cervicalis to marginal mandibular nerve transfer to restore tone and symmetry to the lower third of the face. We have found this a safe and feasible technique with minimal donor site morbidity. The anatomy is favorable both in terms of donor nerve length and size match between nerves. We report 9 cases with at least 6 months follow up which demonstrate improved lower lip tone and symmetry and improved oral competence. Early donor site morbidity and postoperative complications are minimal. This method is a reliable adjunct in facial nerve reconstruction to address the marginal mandibular nerve paralysis.

## Résumé

Une paralysie du nerf facial affectant le bas du visage peut compromettre des fonctions essentielles, comme la parole et l’alimentation. L’asymétrie associée du visage peut contribuer à l’isolement social. La reconstruction du nerf facial au moyen d’une combinaison d’interposition de greffes nerveuses et/ou de transferts de nerfs procure une amélioration significative. Toutefois, ces options sont limitées par le nombre insuffisant de nerfs donneurs et le risque de syncinésies non souhaitées si un seul nerf donneur est utilisé pour toute la moitié du visage. Pour améliorer ces deux limites, nous pratiquons un transfert nerveux de l’anse cervicale vers le nerf mandibulaire marginal pour restaurer le tonus et la symétrie du tiers inférieur du visage. Nous avons trouvé que cette technique est sécuritaire et faisable avec une morbidité minimale au site donneur. L’anatomie est favorable pour ce qui concerne, à la fois, la longueur du nerf donneur et la correspondance de taille entre les nerfs. Nous décrivons neuf cas avec un suivi d’au moins 6 mois qui démontrent l’amélioration du tonus et de la symétrie de la lèvre inférieure, ainsi que l’amélioration de la compétence de la bouche. La morbidité précoce au site donneur et les complications postopératoires sont minimes. Cette méthode est un complément fiable à la reconstruction du nerf facial pour résoudre la paralysie du nerf mandibulaire marginal.

## Introduction

Facial nerve palsy affecting the lower face may compromise essential functions such as speech and eating. The associated facial asymmetry can contribute to social isolation. Facial nerve reconstruction using a combination of interposition nerve grafts and/or nerve transfers offers significant improvement.^1^ However, these options are limited by insufficient donor nerves and unwanted synkinesis if a single donor nerve is used for the entire hemi-face. To ameliorate both limitations, we perform an ansa cervicalis to marginal mandibular nerve transfer to restore tone and symmetry to the lower third of the face. We have found this a safe and feasible technique with minimal donor site morbidity. The anatomy is favourable both in terms of donor nerve length and size match between nerves. We report 9 cases with at least 6-month follow-up, which demonstrate improved lower lip tone and symmetry and improved oral competence. Early donor site morbidity and postoperative complications are minimal. This method is a reliable adjunct in facial nerve reconstruction to address the marginal mandibular nerve paralysis.

An innovative nerve transfer for lower lip facial reanimation is presented whereby the superior root of the ansa cervicalis is transferred to the marginal mandibular branch of the facial nerve with the goal of improving lower lip tone and symmetry, as well as improving oral competence. This approach has not been well described in the existing literature.^2–4^ A major advantage of this technique compared to the well-described hypoglossal-to-facial nerve transfer is the preservation of lingual function.^5–7^

## Anatomy

**Diagram 1.**
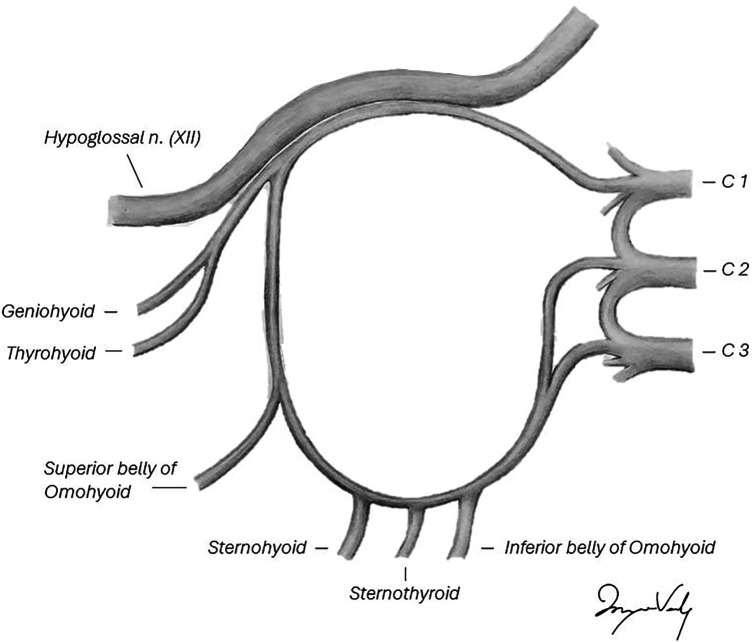
Ansa cervicalis anatomy. Legend: Anatomical illustration of the ansa cervicalis origin, course, and branches.

The ansa cervicalis has a dual origin that forms a nerve loop.^8–10^ The superior root originates from the C1 ventral ramus, travels with the hypoglossal nerve, then leaves to descend on the carotid sheath, giving off a motor branch to the superior belly of the omohyoid muscle.^9,10^ The inferior root originates from the C2 and C3 ventral rami, which descends to the level of the origin of the occipital artery, where it joins the superior root via the ansa cervicalis proper.^11^ This area of the ansa cervicalis gives off motor branches to the sternothyroid, sternohyoid, and inferior belly of the omohyoid muscles^9,10^ (see Diagram 1). The infrahyoid muscles play an active role in swallowing through movement of the larynx.^12^ The thyrohyoid muscle is not innervated by the ansa cervicalis and, therefore, helps preserve the function of swallowing.^12^ The morbidity of a transection of the ansa cervicalis is thought to be minimal.^2,13,14^

## Technique Description

**Figure 1.**
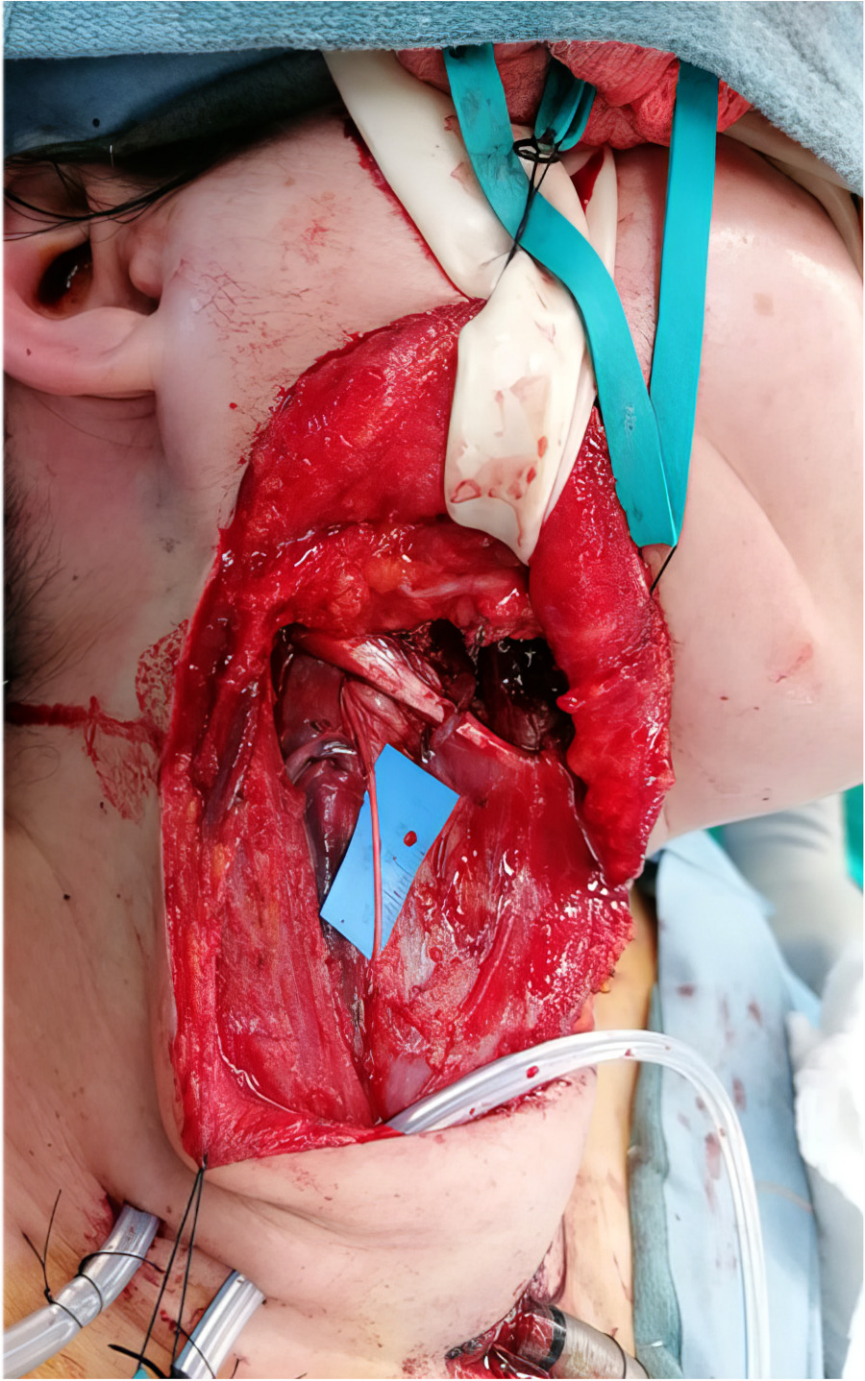
Ansa cervicalis dissection. Legend: The superior root of the ansa cerviclis nerve can be found running in a vertical direction, in close relation to the sternocleidomastoid muscle.

An extended facelift incision that incorporates a transverse neck crease is used, and skin flaps are elevated deep to the superficial musculoaponeurotic system (SMAS) and platysma. The marginal mandibular branch of the facial nerve is identified at the anterior-inferior border of the parotid gland in conjunction with buccal and zygomatic branches. The anterior border of the sternocleidomastoid muscle is identified and reflected laterally to expose the superficial border of the internal jugular vein, which is then exposed in a cephalad direction. Dissection is continued between the sternocleidomastoid muscle and internal jugular vein, and here the superior ramus of the ansa cervicalis can be identified running in a vertical direction (see Figure 1). The superior root is found in close relation to the internal jugular vein.^8,15^ A handheld nerve stimulator confirms that stimulation of the superior ramus is associated with activation of the infrahyoid muscles. The superior ramus is then followed to its take off from the inferior border of the hypoglossal nerve. Once an adequate length of the proposed nerve transfer is ascertained, the ansa cervicalis nerve is divided caudally. The inferior root is left in continuity and thus may still provide nerve input to the sternothyroid, sternohyoid, and inferior belly of the omohyoid muscles. The length is sufficient,^4^ and the diameter a suitable match for the marginal mandibular branch of the facial nerve (see Supplemental Video 1 [Accessible online at https://journals.sagepub.com/doi/full/10.1177/22925503251386749]). If the intention is to harvest the ansa cervicalis at the time of oncologic neck dissection in the setting of head and neck cancer, it is important to communicate to the ablative surgeon the need to preserve the full length of the ansa cervicalis.

## Results

Nine ansa cervicalis to marginal mandibular nerve transfers were performed between 2021 and 2024 (see Table 1). Mandibular nerve palsy was part of a complete facial nerve palsy in all cases; that is, we did not do isolated marginal mandibular nerve palsy cases. In all cases, the nerve transfer was anatomically possible, and the nerve repair was completed without tension. In all cases, the ansa cervicalis was a good match to the marginal mandibular nerve in terms of size. No early postoperative complications or donor site morbidities were documented in any of the 9 patients.

**Table 1.** Results.

	Patient demographics						Outcomes				
	Age at surgery (years)	Co-morbidities	Diagnosis	Other facial nerve procedures	Timing of surgery	Follow-up assessment (months)	FaCE		HB		SB
							Total score	Oral function	Overall function	MM function	Overall function
1	39	S	Schwannoma	VNG and MFNT	Immediate	40	61	100	3	2	34
2	54	HLD	Schwannoma	CFNG and MFNT	Delayed	44	56	88	4	2	41
3	68	HPT, Gout, and HLD	Schwannoma	CFNG, MFNT, and GW	Delayed	14	36	75	4	3	39
4	52	HPT, BMI, BA, and GERD	Schwannoma	CFNG and MFNT	Immediate	31	63	75	4	2	45
5	58	S, CHF, RF, OSA, and HLD	Schwannoma	VNG	Immediate	28	67	100	4	2	47
6	66	None	Facial spasms	MFNT	Immediate	7	41	25	4	2	12
7	22	S, Hep, and BA	Lymphoepithelial carcinoma	VNG, MFNT, and GW	Immediate	7	63	88	4	3	32
8	48	None	Schwannoma	VNG and MFNT	Immediate	6	39	63	3	2	16
9	55	OA and TIA	Schwannoma	CFNG and MFNT	Delayed	6	36	63	4	3	16

Patients were evaluated at a minimum of 6 months postoperatively using the FaCE scale, the Sunnybrook Facial Grading System, and the House-Brackmann Facial Paralysis Scale.

Abbreviations: S, smoker; HLD, hyperlipidemia; HPT, hypertension; BMI, high body mass index; BA, bronchial asthma; GERD, gastro-esophegeal reflux disease; CHF, congestive heart failure; RF, renal failure; OSA, obstructive sleep apnea; Hep, hepatitis; OA, osteoarthritis; TIA, transient ischemic attack; VNG, vascularized nerve graft; CFNG, cross-facial nerve graft; MFNT, Masseteric to facial nerve transfer; GW, gold weight; FaCE, Facial Clinimetric Evaluation scale instrument; HB, House-Brackmann Facial Paralysis Scale; MM, Marginal mandibular nerve; SFGS, Sunnybrook facial grading system.

All 9 patients were seen for a follow-up evaluation at a minimum of 6 months postoperatively. We assessed patients using the Facial Clinimetric Evaluation (FaCE) scale,^16^ the Sunnybrook Facial Grading System (SB),^17^ and the House-Brackmann Facial Paralysis Scale (HB).^18^ Both the FaCe scale and the SB were scored out of 100, with 100 being normal facial function.^16,19^ The HB is scored from 1 to 6, with 1 being normal facial function.^18^ To help assess the lower lip more directly, we calculated an overall facial score using the FaCe scale as well as an oral function score in isolation. As shown in Table 1, 8 out of 9 patients scored above 60 with the oral function component, and of these, 4 patients scored above 80. When using the HB scale, we scored the overall facial function as well as the marginal mandibular function in isolation. All patients scored 3 or lower when the marginal mandibular branch was evaluated in isolation. All patients exhibited good function of the marginal mandibular branch using SB, with good lower lip tone and oral competence.

**Figure 2.**
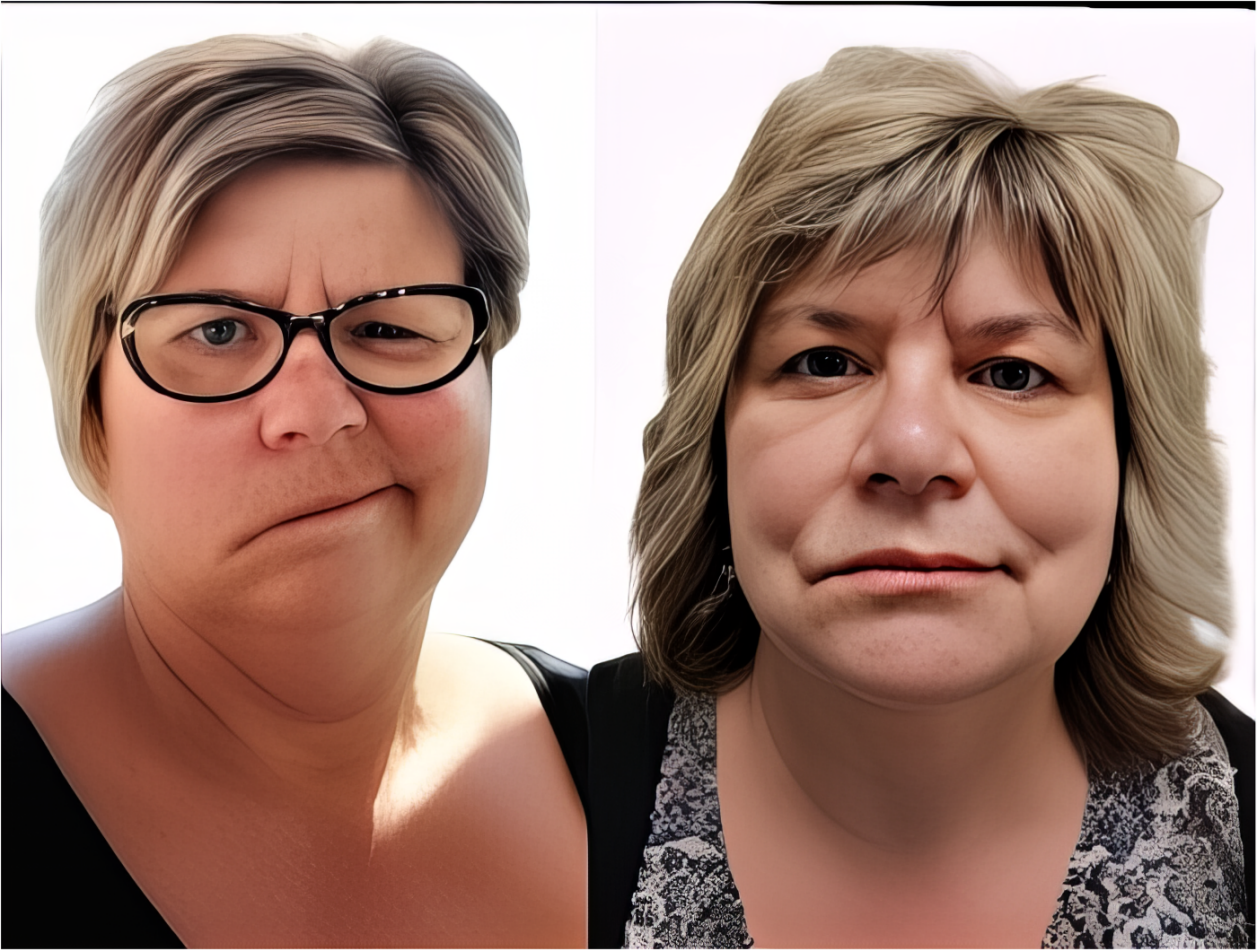
Pre- and postoperative photographs. Legend: Pre- and postoperative images of a patient receiving ansa cervicalis to marginal mandibular nerve transfer, cross-face nerve graft, and masseter to buccal branch nerve transfers demonstrate good improvement.

Pre- and postoperative images of a patient receiving ansa cervicalis to marginal mandibular nerve transfer, cross-face nerve graft, and masseter to buccal branch nerve transfers demonstrate good improvement (see Figure 2). A major improvement is seen in symmetry and lower lip tone. The lower lip height is similar to the normal side position when compared to the preoperative photograph.

Electromyography demonstrates that lower lip musculature is innervated via the nerve transfer (see Supplemental Video 2 [Accessible online at https://journals.sagepub.com/doi/full/10.1177/22925503251386749]). The lower lip is activated by the swallow activation. Notably, no patient complained of donor site concerns.

## Discussion

Facial nerve palsy is a debilitating state in which the essential functions of eating, speaking, and expressing emotions are compromised.^20–22^ Most efforts for reconstruction have been focused on the periorbital and midface areas to protect the eye from excessive exposure to the external environment and to create a socially acceptable smile.^23,24^ The marginal mandibular branch is often neglected in facial nerve reconstruction, even though its paralysis can result in asymmetry and drooping of the lower lip, which may in turn affect speech, oral competence, and emotional expression.^23,24^

This unique technique of lower lip facial reanimation, using a nerve transfer from the superior ramus of the ansa cervicalis to the marginal mandibular nerve, can provide lower lip tone and symmetry, and thereby improve oral competence. Reinnervation occurs between 5 and 6 months, demonstrated clinically as improved commissure and lower lip position or by EMG study.

Ansa to marginal mandibular nerve transfer is now standard of care in Calgary for complete facial nerve reconstruction in young patients presenting at < 9 months from inciting paralyzing event, as it is associated with few negative consequences and helps lower lip position and function.

## Limitations

This retrospective study is limited by the absence of a control group and the lack of formal preoperative functional assessment. In all cases, the marginal mandibular nerve was completely transected, and function was therefore assumed to be zero. Further prospective studies with appropriate control groups are planned to evaluate our results more objectively and strengthen the evidence base.

## Supplemental Material

**Figure:** Video 1. Technique description Legend : Step by step guide to the ansa cervicalis to marginal mandibular nerve transfer

**Figure:** Video 2. Electromyography evaluation Legend: EMG demonstrates that lower lip musculature is innervated via the nerve transfer
